# Multiscale study of memory type simple ratio estimators in two stage sampling under exponentially weighted moving averages

**DOI:** 10.1371/journal.pone.0335586

**Published:** 2025-11-13

**Authors:** Kanwal Shafiq Minhas, Hatem E. Semary, Riffat Jabeen, Azam Zaka

**Affiliations:** 1 Department of Statistics, COMSATS University Islamabad, Lahore Campus, Lahore, Pakistan; 2 Department of Mathematics and Statistics, College of Science, Imam Mohammad Ibn Saud Islamic University (IMSIU), Riyadh, Saudi Arabia; 3 Department of Statistics, Government Graduate College of Science, Lahore, Pakistan; RMIT University, AUSTRALIA

## Abstract

Two-stage cluster sampling is often employed in survey sampling when complete population information is not available. In this setting, the Exponentially Weighted Moving Average (EWMA) statistic offers an efficient way to estimate the population mean by incorporating both past and current data. Motivated by this, we propose a class of memory-type ratio and exponential estimators for estimating the population mean under a two-stage cluster sampling framework. Theoretical expressions for the biases and mean square errors (MSE) of the proposed estimators are derived. To evaluate their performance, a comprehensive simulation study was carried out, supplemented by an empirical application. Several special cases of the proposed estimators were also considered and compared with existing two-stage estimators. The analysis was performed under different values of the EWMA smoothing constant (λ=0.3,0.5,0.75,0.9). Both simulation and empirical results consistently show that the proposed memory-type two-stage ratio estimators outperform existing approaches, providing improved efficiency with minimum MSE.

## 1. Introduction

In survey sampling, when population information is not fully available, the cluster sampling technique is often ideal for selecting a random sample. When this procedure is implemented in two stages, it becomes two-stage cluster sampling [1]. In this design, a random sample of nnn clusters, referred to as first-stage units (FSUs), is selected from the total of *N* clusters. Subsequently, from each of the nnn selected clusters of size *M*_*i*_ (*i = 1, 2… n*), a random sample of *m*_*i*_ elements is drawn, known as the second-stage units (SSUs) [2].

[3] suggested general families of factor-type estimators for the population mean under the condition that non-response occurs either on the study variable alone or on both the study and auxiliary variables, making use of auxiliary variable information. Further [4], recommended calibration based estimation of finite population total in two stage random sampling. [5,6] proposed generalized ratio estimators using single and two auxiliary variables in two-stage sampling and exponential estimators to estimate population mean using two-phase scheme of sampling considering two auxiliary variables in two-stage sampling. The problem of estimating population mean in the presence of non-response under two stage technique of sampling was suggested by [7]. By using [8] technique, the estimator under two non-response models has been established. Moreover [9], proposed five different two-stage sampling designs based on neoteric ranked set sampling (NRSS). In another study [10], introduced a new two-stage cluster sampling design in which the first-stage sampling was carried out using probability proportional to size (PPS) and the second stage employed RSS to address variability in population unit sizes at the first stage. Similarly [11], analyzed clustered data under two-stage informative sampling within two-level models. Their investigation provided a new method to estimate cluster effects while accounting for design weights using a normal approximation. Furthermore [12], investigated a general class of exponential and chain ratio-type estimators for estimating the population mean under two-stage sampling with two auxiliary variables in the presence of measurement error. In addition [13], proposed a new generalized class of estimators based on PPS sampling with two auxiliary variables for estimating the finite population mean.

Several studies have extended memory-type estimators using the EWMA statistic in different sampling settings. For time-based surveys, memory-type ratio and product estimators were proposed in [14–16], while their application under ranked-based sampling (RBS) schemes such as extreme, median, and quartile RSS was also investigated. A hybrid EWMA approach was introduced in [17] to improve process mean estimation using secondary information. Further extensions include memory-type estimators with auxiliary variables in two-phase simple random sampling [18], logarithmic estimators using EWMA [19], and hybrid EWMA-based logarithmic estimators [20]. Variance estimation in the presence of measurement errors was addressed in [21] through memory-type ratio and product estimators with EWMA. Additional developments include the use of auxiliary variables with extended EWMA statistics [22] and optimal classes of memory-type simple ratio estimators in two-stage sampling [23].

Several extensions of the EWMA statistic have been proposed in recent studies. A homogeneously weighted moving average chart for variance was developed in [24] for rapid detection of process shifts using auxiliary information, while [25] introduced an adaptive EWMA-based control chart for the coefficient of variation under time-varying process means and linearly changing standard deviations. In healthcare applications [26], addressed patient risk adjustment by designing EWMA charts based on residuals from support vector machine regression, and [27] extended this idea to monitoring process dispersion across diverse environments through adaptive EWMA charts using support vector regression. For survey estimation [28], proposed generalized families of exponential estimators based on EWMA, while [29] introduced optimal memory-type methods for imputing missing time-based survey data. Furthermore [30], analyzed the effects of measurement and non-response errors on estimation accuracy, considering cases where non-response affected only the study variable or both study and auxiliary variables.

Several studies have examined two-stage sampling and memory-type estimators using different statistics. Building on this literature, the present study develops ratio and exponential estimators under a two-stage sampling scheme by incorporating the EWMA statistic for time-scale surveys at the second stage. The memory-type estimator integrates both past and present information through the EWMA approach introduced by [16]. Specifically, we propose generalized two-stage simple ratio estimators for three cases: (i) unequal first-stage units with weighted mean, (ii) unequal first-stage units with unweighted mean, and (iii) equal-sized first-stage units. To evaluate their performance, a simulation study is conducted across these scenarios.

The remainder of this paper is organized as follows. Section 2 reviews existing two-stage ratio and exponential estimators under different cases of first-stage unit selection. Section 3 introduces the proposed memory-type ratio estimators using the EWMA statistic and derives their bias and MSE, while Section 4 develops the corresponding exponential estimators. Section 5 presents a simulation study comparing the proposed methods with existing ones, and Section 6 illustrates their application using international trade data. Finally, Section 7 concludes with key findings and possible future directions.

## 2. Existing two stage estimators

The brief detail of some existing ratio and two stage sampling estimators for the specified three cases are

### 2.1. Sukhatme et al. (1984)—Two stage estimator

#### 2.1.1. Case-I.

The two stage estimator [31] is given below when FSU are unequal and weighted mean is used


$$\begin{array}{c} T_{\text{1}}=\frac{\text{1}}{n}\sum {\mathrm{\backslash nolimits}}_{i=\text{1}}^{n}\eta_{i}{\overset{―}{y}}_{i}, \end{array}$$ (1)

the Mean Square Error (MSE) as


$$\begin{array}{c} MSE\left(T_{\text{1}}\right)=\left(\frac{\text{1}}{n}-\frac{\text{1}}{N}\right){S_{yt}^{o}}^{\text{2}}+\frac{\text{1}}{nN}\sum {\mathrm{\backslash nolimits}}_{i=\text{1}}^{N}{\eta_{i}}^{\text{2}}\left(\frac{\text{1}}{m_{i}}-\frac{\text{1}}{M_{i}}\right){S_{yi}^{o}}^{\text{2}}. \end{array}$$ (2)

#### 2.1.2. Case-II.

The two stage estimator is given below when FSU are unequal and unweighted mean is used


$$\begin{array}{c} T_{\text{2}}=\frac{\text{1}}{n}\sum {\mathrm{\backslash nolimits}}_{i=\text{1}}^{n}{\overset{―}{y}}_{i}, \end{array}$$ (3)

the MSE of the estimator can be as follows


$$\begin{array}{c} MSE\left(T_{\text{2}}\right)=\left(\frac{\text{1}}{n}-\frac{\text{1}}{N}\right){S_{yt}^{*}}^{\text{2}}+\frac{\text{1}}{nN}\sum {\mathrm{\backslash nolimits}}_{i=\text{1}}^{N}\left(\frac{\text{1}}{m_{i}}-\frac{\text{1}}{M_{i}}\right){S_{yi}^{*}}^{\text{2}}. \end{array}$$ (4)

#### 2.1.3. Case-III.

The two stage estimator when FSU are of equal size is


$$\begin{array}{c} T_{\text{3}}=\frac{\text{1}}{n}\sum {\mathrm{\backslash nolimits}}_{i=\text{1}}^{n}\eta_{i}{\overset{―}{y}}_{i}, \end{array}$$ (5)

the MSE for Case-III as


$$\begin{array}{c} MSE\left(T_{\text{3}}\right)=\left(\frac{\text{1}}{n}-\frac{\text{1}}{N}\right){S_{yt}^{`}}^{\text{2}}+\frac{\text{1}}{nN}\sum {\mathrm{\backslash nolimits}}_{i=\text{1}}^{N}\left(\frac{\text{1}}{m_{i}}-\frac{\text{1}}{M_{i}}\right){S_{yi}^{`}}^{\text{2}}. \end{array}$$ (6)

### 2.2. Sukhatme et al. (1984)—Two stage ratio estimators

#### 2.2.1. Case-I.

The two stage estimator when FSU are unequal and weighted mean is used can be written as


$$T_{\text{4}}=\frac{{\overset{―}{t}}_{\text{1}s}}{{\overset{―}{u}}_{\text{1}s}}{\overset{―}{U}}_{\text{1}s}$$ (7)

the MSE can be inscribed as


$$\begin{array}{c} MSE\left(T_{\text{4}}\right)\approx \left(\frac{\text{1}}{n}-\frac{\text{1}}{N}\right)\left\{S_{\text{1}bt}^{\text{2}}-\text{2}RS_{\text{1}btu}+R^{\text{2}}S_{\text{1}bu}^{\text{2}}\right\}+\frac{\text{1}}{nN}\sum {\mathrm{\backslash nolimits}}_{i=\text{1}}^{N}{\eta_{i}}^{\text{2}}\left(\frac{\text{1}}{m_{i}}-\frac{\text{1}}{M_{i}}\right)\left\{S_{\text{1}wti}^{\text{2}}-\text{2}RS_{\text{1}wiut}+R^{\text{2}}S_{\text{1}wui}^{\text{2}}\right\}. \end{array}$$ (8)

#### 2.2.2. Case-II.

The two stage estimator is given below when FSU are unequal and unweighted mean is used


$$T_{\text{5}}=\frac{{\overset{―}{t}}_{\text{2}s}}{{\overset{―}{u}}_{\text{2}s}}{\overset{―}{U}}_{\text{2}s},$$ (9)

the MSE can be as follows


$$\begin{array}{c} MSE\left(T_{\text{5}}\right)\approx \left(\frac{\text{1}}{n}-\frac{\text{1}}{N}\right)\left\{S_{\text{2}bt}^{\text{2}}-\text{2}RS_{\text{2}btu}+R^{\text{2}}S_{\text{2}bu}^{\text{2}}\right\}+\frac{\text{1}}{nN}\sum {\mathrm{\backslash nolimits}}_{i=\text{1}}^{N}{\eta_{i}}^{\text{2}}\left(\frac{\text{1}}{m_{i}}-\frac{\text{1}}{M_{i}}\right)\left\{S_{\text{2}wti}^{\text{2}}-\text{2}RS_{\text{2}wiut}+R^{\text{2}}S_{\text{2}wui}^{\text{2}}\right\}. \end{array}$$ (10)

#### 2.2.3. Case-III.

The two stage estimator when FSU are of equal size is


$$T_{\text{6}}=\frac{{\overset{―}{t}}_{\text{3}s}}{{\overset{―}{u}}_{\text{3}s}}{\overset{―}{U}}_{\text{3}s},$$ (11)

the MSE of the estimator is


$$\begin{array}{c} MSE\left(T_{6}\right)\approx \left(\frac{1}{n}-\frac{1}{N}\right)\left\{S_{3bt}^{2}-2RS_{3btu}+R^{2}S_{3bu}^{2}\right\}+\frac{1}{nN}\sum {\mathrm{\backslash nolimits}}_{i=1}^{N}{\eta_{i}}^{2}\left(\frac{1}{m_{i}}-\frac{1}{M_{i}}\right)\left\{S_{3wti}^{2}-2RS_{3wiut}+R^{2}S_{3wui}^{2}\right\}. \end{array}$$ (12)

## 3. Proposed simple ratio estimators in two stage sampling using EWMA statistic

In this section, generalized memory type ratio estimators have been recommended for two stage sampling using [31].

### 3.1. Notations

#### 3.1.1. Case-I: Equal FSU and weighted mean is used.

Let a population consists of *N* called FSU and each FSU comprises of *M*_*i*_ named as SSU. Assume a sample of *n* FSU and a sample of *m*_*i*_ SSU from each of *n* FSU selected by allocating the weights $\left(\eta_{i}=\frac{M_{i}}{\overset{―}{M}}\right)$ to the SSU respectively. Suppose that the selection of units at each stage has been done using simple random sampling. The following notations are used to obtain the proposed estimators.

$t_{ij}$ is the value of *j*^*th*^ SSU in the *i*^*th*^ FSU *(j = 1, 2…, M & i = 1, 2…, N).*

${\overset{―}{T}}_{i}=\frac{\text{1}}{M_{i}}\sum_{j=\text{1}}^{M_{i}}t_{ij}$ & ${\overset{―}{t}}_{i}=\frac{\text{1}}{m_{i}}\sum_{j=\text{1}}^{m_{i}}t_{ij}$ are the means per SSU in the *i*^*th*^ FSU in population and sample respectively.

${\overset{―}{T}}_{s}=\frac{\text{1}}{N}\sum_{i=\text{1}}^{N}\frac{\text{1}}{\overset{―}{M}}\sum_{j=\text{1}}^{M_{i}}M_{i}{\overset{―}{t}}_{i}\text{\textbackslash{} and}{\overset{―}{t}}_{s}=\frac{\text{1}}{n}\sum_{i=\text{1}}^{n}\frac{M_{i}}{\overset{―}{M}}{\overset{―}{t}}_{i}$ = the mean per SSU in population and sample.

$\overset{―}{M}=\frac{\sum_{i=\text{1}}^{N}M_{i}}{N}$ is the average of SSU in population.

${\overset{―}{u}}_{i}=\frac{\text{1}}{m_{i}}\sum_{j=\text{1}}^{m_{i}}u_{ij}$, denotes the mean per SSU in the *i*^*th*^ FSU in the sample.

${\overset{―}{u}}_{s}=\frac{\text{1}}{n}\sum_{i=\text{1}}^{n}\frac{M_{i}}{\overset{―}{M}}{\overset{―}{u}}_{i}$ is the mean per SSU in sample.

$u_{ij}=\lambda \overset{―}{x}+(\text{1}-\lambda )u_{ij-\text{1}}$ represents the EWMA statistic per SSU in the sample for auxiliary information.

$t_{ij}=\lambda \overset{―}{y}+(\text{1}-\lambda )t_{ij-\text{1}}$ is the EWMA statistic for study variable per SSU in the sample.

Let us consider, ${\overset{―}{t}}_{s}={\overset{―}{T}}_{s}\left(\text{1}+e_{o}\right),{\overset{―}{u}}_{s}={\overset{―}{U}}_{s}\left(\text{1}+e_{\text{1}}\right)$ and the respective expectations are


$$\begin{array}{c} \begin{array}{c} E\left(e_{o}\right)=E\left(e_{\text{1}}\right)=\text{0}\\ E\left(e_{o}^{\text{2}}\right)=var\left(r_{\text{1}}\right)=\frac{\text{1}}{{\overset{―}{T}}_{s}^{\text{2}}}\left\{\left(\frac{\text{1}}{n}-\frac{\text{1}}{N}\right)S_{bt}^{\text{2}}+\frac{\text{1}}{nN}\sum_{i=\text{1}}^{N}{\eta_{i}}^{\text{2}}\left(\frac{\text{1}}{m_{i}}-\frac{\text{1}}{M_{i}}\right)S_{wti}^{\text{2}}\right\}\\ E\left(e_{\text{1}}^{\text{2}}\right)=var\left(r_{\text{2}}\right)=\frac{\text{1}}{{\overset{―}{U}}_{s}^{\text{2}}}\left\{\left(\frac{\text{1}}{n}-\frac{\text{1}}{N}\right)S_{bu}^{\text{2}}+\frac{\text{1}}{nN}\sum_{i=\text{1}}^{N}{\eta_{i}}^{\text{2}}\left(\frac{\text{1}}{m_{i}}-\frac{\text{1}}{M_{i}}\right)S_{wui}^{\text{2}}\right\} \end{array}\\ E\left(e_{o}e_{\text{1}}\right)=var(r_{\text{3}})=\frac{\text{1}}{{\overset{―}{T}}_{s}{\overset{―}{U}}_{s}}\left\{\left(\frac{\text{1}}{n}-\frac{\text{1}}{N}\right)\rho_{btu}S_{bt}S_{bu}+\frac{\text{1}}{nN}\sum_{i=\text{1}}^{N}{\eta_{i}}^{\text{2}}\left(\frac{\text{1}}{m_{i}}-\frac{\text{1}}{M_{i}}\right)\rho_{wiut}S_{iwt}S_{ibu}\right\} \end{array}],$$ (13)

where, $\begin{array}{c} S_{bt}^{\text{2}}=\frac{\text{1}}{N-\text{1}}\sum_{i=\text{1}}^{N}{\left(t_{ij}-{\overset{―}{T}}_{s}\right)}^{\text{2}},S_{wti}^{\text{2}}=\frac{\text{1}}{M_{i}-\text{1}}\sum_{i=\text{1}}^{n}{\left(t_{ij}-{\overset{―}{T}}_{i}\right)}^{\text{2}},C_{bt}=\frac{S_{bt}^{\text{2}}}{{\overset{―}{T}}_{s}},C_{bu}=\frac{S_{bu}^{\text{2}}}{{\overset{―}{U}}_{s}},C_{wti}=\frac{S_{wti}^{\text{2}}}{{\overset{―}{T}}_{s}},C_{wiu}=\frac{S_{wiu}^{\text{2}}}{{\overset{―}{U}}_{s}},\rho_{btu}=\frac{S_{btu}}{S_{bt}S_{bu}}\rho_{tiwu}= \end{array}$ $\begin{array}{c} \frac{S_{iwtu}}{S_{iwt}S_{iwu}}. \end{array}$

#### 3.1.2. Case-II: Unequal FSU and unweighted mean is used.

For case-II, assume the equal weights for all unequal FSU to obtain an unweighted and biased estimator of population mean in two-stage sampling plan. The procedure for the two stage sampling design in this situation is the same as explained previously in case-I. The notations and expectations for this case can be defined as

$\eta_{i}=\frac{M_{i}}{\overset{―}{M}}=\text{1}$

$\begin{array}{c} {\overset{―}{T}}_{s}^{,}=\frac{\text{1}}{N}\sum_{i=\text{1}}^{N}{\overset{―}{t}}_{i} \end{array}$ is the mean per SSU in population.

${\overset{―}{t}}_{s}^{,}=\frac{\text{1}}{n}\sum_{i=\text{1}}^{n}{\overset{―}{t}}_{i}$ is the mean per SSU in sample.

Now, let ${\overset{―}{t}}_{s}^{,,}={\overset{―}{T}}_{s}^{,}\left(\text{1}+e_{o}^{,}\right),{\overset{―}{u}}_{s}^{,}={\overset{―}{U}}_{s}^{,}\left(\text{1}+e_{\text{1}}^{,}\right)$ and the respective expectations are


$$\begin{array}{c} \begin{array}{c} E\left(e_{o}^{,}\right)=E\left(e_{\text{1}}^{,}\right)=\text{0}\\ E\left(e_{o}^{,\text{2}}\right)=var\left(r_{\text{1}}^{,}\right)=\frac{\text{1}}{{\overset{―}{T}}_{s}^{,\text{2}}}\left\{\left(\frac{\text{1}}{n}-\frac{\text{1}}{N}\right)S_{bt}^{,\text{2}}+\frac{\text{1}}{nN}\sum_{i=\text{1}}^{N}{\eta_{i}}^{\text{2}}\left(\frac{\text{1}}{m_{i}}-\frac{\text{1}}{M_{i}}\right)S_{wti}^{,\text{2}}\right\}\\ E\left(e_{\text{1}}^{,\text{2}}\right)=var\left(r_{\text{2}}^{,}\right)=\frac{\text{1}}{{\overset{―}{U}}_{s}^{,\text{2}}}\left\{\left(\frac{\text{1}}{n}-\frac{\text{1}}{N}\right)S_{bu}^{,\text{2}}+\frac{\text{1}}{nN}\sum_{i=\text{1}}^{N}{\eta_{i}}^{\text{2}}\left(\frac{\text{1}}{m_{i}}-\frac{\text{1}}{M_{i}}\right)S_{wui}^{,\text{2}}\right\} \end{array}\\ E\left(e_{o}^{,}e_{\text{1}}^{,}\right)=var(r_{\text{3}}^{,})=\frac{\text{1}}{{\overset{―}{T}}_{s}^{,}{\overset{―}{U}}_{s}^{,}}\left\{\left(\frac{\text{1}}{n}-\frac{\text{1}}{N}\right)\rho_{btu}^{,}S_{bt}^{,}S_{bu}^{,}+\frac{\text{1}}{nN}\sum_{i=\text{1}}^{N}{\eta_{i}}^{\text{2}}\left(\frac{\text{1}}{m_{i}}-\frac{\text{1}}{M_{i}}\right)\rho_{wiut}^{,}S_{iwt}^{,}S_{iwu}^{,}\right\} \end{array}],$$ (14)

where, $\mathrm{\backslash footnotesize}\begin{array}{c} S_{bt}^{,\text{2}}=\frac{\text{1}}{N-\text{1}}\sum_{i=\text{1}}^{N}{\left(t_{ij}-{\overset{―}{T}}_{s}^{,}\right)}^{\text{2}},S_{wti}^{,\text{2}}=\frac{\text{1}}{M_{i}-\text{1}}\sum_{i=\text{1}}^{n}{\left(t_{ij}-{\overset{―}{T}}_{i}\right)}^{\text{2}},C_{bt}^{,}=\frac{S_{bt}^{,\text{2}}}{{\overset{―}{T}}_{s}^{,}},C_{bu}^{,}=\frac{S_{bu}^{,\text{2}}}{{\overset{―}{U}}_{s}^{,}},C_{wti}^{,}=\frac{S_{wti}^{,\text{2}}}{{\overset{―}{T}}_{s}^{,}},C_{wiu}^{,}=\frac{S_{wui}^{,\text{2}}}{{\overset{―}{U}}_{s}^{,}},\rho_{btu}^{,}=\frac{S_{btu}^{,}}{S_{bt}^{,}S_{bu}^{,}}\&\rho_{wiut}^{,}=\frac{S_{iwtu}^{,}}{S_{iwt}^{,}S_{iwu}^{,}}. \end{array}$

#### 3.1.3. Case-III: Equal sizes FSU.

Suppose that a population comprises of *N* FSU and each FSU consists of *M*, SSU and let a sample of *n* FSU is selected and a sample of *m* SSU from each of *n* FSU. Assume that the units have been selected through simple random sampling technique. To obtain the expressions of biases and MSE’s under two stage random sampling, the following symbolizations is used. Consider,

${\overset{―}{T}}_{s}^{,,}=\frac{\text{1}}{N}\sum_{i=\text{1}}^{N}{\overset{―}{t}}_{i}$ is the mean per SSU in population

${\overset{―}{t}}_{s}^{,,}=\frac{\text{1}}{n}\sum_{i=\text{1}}^{n}{\overset{―}{t}}_{i}$ is the mean per SSU in sample.

Now, let ${\overset{―}{t}}_{s}^{,,}={\overset{―}{T}}_{s}^{,,}\left(\text{1}+e_{o}^{,,}\right),{\overset{―}{u}}_{s}^{,,}={\overset{―}{U}}_{s}^{,}\left(\text{1}+e_{\text{1}}^{,,}\right)$ and the respective expectations are


$$\begin{array}{c} \begin{array}{c} E\left(e_{o}^{,,}\right)=E\left(e_{\text{1}}^{,,}\right)=\text{0}\\ E\left(e_{o}^{,,\text{2}}\right)=var\left(r_{\text{1}}^{,,}\right)=\frac{\text{1}}{{\overset{―}{T}}_{s}^{,,\text{2}}}\left\{\left(\frac{\text{1}}{n}-\frac{\text{1}}{N}\right)S_{bt}^{,,\text{2}}+\frac{\text{1}}{nN}\sum_{i=\text{1}}^{N}{\eta_{i}}^{\text{2}}\left(\frac{\text{1}}{m_{i}}-\frac{\text{1}}{M_{i}}\right)S_{wti}^{,,\text{2}}\right\}\\ E\left(e_{\text{1}}^{,,\text{2}}\right)=var\left(r_{\text{2}}^{,,}\right)=\frac{\text{1}}{{\overset{―}{U}}_{s}^{,,\text{2}}}\left\{\left(\frac{\text{1}}{n}-\frac{\text{1}}{N}\right)S_{bu}^{,,\text{2}}+\frac{\text{1}}{nN}\sum_{i=\text{1}}^{N}{\eta_{i}}^{\text{2}}\left(\frac{\text{1}}{m_{i}}-\frac{\text{1}}{M_{i}}\right)S_{wui}^{,,\text{2}}\right\} \end{array}\\ E\left(e_{o}^{,,}e_{\text{1}}^{,,}\right)=var(r_{\text{3}}^{,,})=\frac{\text{1}}{{\overset{―}{T}}_{s}^{,,}{\overset{―}{U}}_{s}^{,,}}\left\{\left(\frac{\text{1}}{n}-\frac{\text{1}}{N}\right)\rho_{btu}^{,,}S_{bt}^{,,}S_{bu}^{,,}+\frac{\text{1}}{nN}\sum_{i=\text{1}}^{N}{\eta_{i}}^{\text{2}}\left(\frac{\text{1}}{m_{i}}-\frac{\text{1}}{M_{i}}\right)\rho_{wiut}^{,,}S_{iwt}^{,,}S_{iwu}^{,,}\right\} \end{array}],$$ (15)

where, $\mathrm{\backslash footnotesize}\begin{array}{c} S_{bt}^{,,\text{2}}=\frac{\text{1}}{N-\text{1}}\sum_{i=\text{1}}^{N}{\left(t_{ij}-{\overset{―}{T}}_{s}^{,,}\right)}^{\text{2}},S_{wti}^{,,\text{2}}=\frac{\text{1}}{M_{i}-\text{1}}\sum_{i=\text{1}}^{n}{\left(t_{ij}-{\overset{―}{T}}_{i}\right)}^{\text{2}},C_{bt}^{,,}=\frac{S_{bt}^{,,\text{2}}}{{\overset{―}{T}}_{s}^{,,}},C_{bu}^{,,}=\frac{S_{bu}^{,,\text{2}}}{{\overset{―}{U}}_{s}^{,,}},C_{wti}^{,,}=\frac{S_{wti}^{,,\text{2}}}{{\overset{―}{T}}_{s}^{,,}},C_{wiu}^{,,}=\frac{S_{wui}^{,,\text{2}}}{{\overset{―}{U}}_{s}^{,,}},\rho_{btu}^{,,}=\frac{S_{btu}^{,,}}{S_{bt}^{,,}S_{bu}^{,,}}\&\rho_{wiut}^{,,}=\frac{S_{iwtu}^{,,}}{S_{iwt}^{,,}S_{iwu}^{,,}}. \end{array}$

### 3.2. The Bias and MSE for proposed two stage ratio estimators

The suggested ratio estimator for two stage sampling design using memory type at second stage for the following three cases are

#### 3.2.1. Case-I.

The ratio estimator may be defined as


$$R_{ts}=\frac{{\overset{―}{t}}_{s}}{{\overset{―}{u}}_{s}}{\overset{―}{U}}_{s},$$ (16)

using the notations mentioned in section (3.1.1), the estimator in Eq. (16) can be written as


$$R_{ts}={\overset{―}{T}}_{s}\left(\text{1}+e_{o}\right){\left(\text{1}+e_{\text{1}}\right)}^{-\text{1}},$$ (17)

now, expanding the series, ${\left(\text{1}+e_{\text{1}}\right)}^{-\text{1}}$, up to second order, we obtained


$$R_{ts}={\overset{―}{T}}_{s}\left(\text{1}+e_{o}\right)\left(\text{1}-e_{\text{1}}+{e_{\text{1}}}^{\text{2}}\right),$$ (18)

by simplifying the Eq. (18), we get


$$R_{ts}-{\overset{―}{T}}_{s}={\overset{―}{T}}_{s}\left(e_{o}-e_{\text{1}}+{e_{\text{1}}}^{\text{2}}-e_{o}e_{\text{1}}\right),$$ (19)

take expectation on both sides, we obtained the Bias ($R_{ts}$) as


$$Bias\left(R_{ts}\right)={\overset{―}{T}}_{s}\left\{var\left(r_{\text{2}}\right)-var(r_{\text{3}})\right\},$$ (20)

squaring and taking expectation on both sides in Eq. (17), ignoring square and higher terms, we have the MSE expression for the proposed estimator as


$$MSE\left(R_{ts}\right)\approx {{\overset{―}{T}}_{s}}^{\text{2}}\left\{var\left(r_{\text{1}}\right)+var\left(r_{\text{2}}\right)-\text{2}var\left(r_{\text{3}}\right)\right\}.$$ (21)

#### 3.2.2. Case-II.

The ratio estimator in this case can be inscribed as


$$R_{ts}^{,}=\frac{{\overset{―}{t}}_{s}^{,}}{{\overset{―}{u}}_{s}^{,}}{\overset{―}{U}}_{s}^{,}$$ (22)

by utilizing the notations in section (3.1.2), the bias and MSE of estimator given in Eq. (22) may be defined as


$$Bias\left(R_{ts}^{,}\right)={\overset{―}{T}}_{s}^{,}\left\{var\left(r_{\text{2}}^{,}\right)-var(r_{\text{3}}^{,})\right\},$$ (23)

$$MSE\left(R_{ts}^{,}\right)\approx {\overset{―}{T}}_{s}^{,\text{2}}\left\{var\left(r_{\text{1}}^{,}\right)+var\left(r_{\text{2}}^{,}\right)-\text{2}var\left(r_{\text{3}}^{,}\right)\right\}.$$ (24)

#### 3.2.3. Case-III.

The ratio estimator for case-III can be defined as


$$R_{ts}^{,,}=\frac{{\overset{―}{t}}_{s}^{,,}}{{\overset{―}{u}}_{s}^{,,}}{\overset{―}{U}}_{s}^{,,},$$ (25)

by utilizing the notations in section (3.1.3), the bias and MSE of estimator given in Eq. (25) may be defined as


$$Bias\left(R_{ts}^{,,}\right)={\overset{―}{T}}_{s}^{,,}\left\{var\left(r_{\text{2}}^{,,}\right)-var(r_{\text{3}}^{,,})\right\},$$ (26)

$$MSE\left(R_{ts}^{,,}\right)\approx {\overset{―}{T}}_{s}^{,,\text{2}}\left\{var\left(r_{\text{1}}^{,,}\right)+var\left(r_{\text{2}}^{,,}\right)-\text{2}var\left(r_{\text{3}}^{,,}\right)\right\}.$$ (27)

## 4. Proposed exponential estimators in two stage sampling using EWMA statistic

This section introduced the proposed generalized exponential estimators separately by following [32] using single auxiliary variable for three different cases in two stage sampling as mentioned previously.

### 4.1. Case-I

The modified exponential ratio estimator in two stage sampling scheme using memory type estimator can be defined in the form of $e_{o}\text{and}e_{\text{1}}$ as


$$R_{ts}^{\text{1}}={\overset{―}{t}}_{s}exp\left[\frac{{\overset{―}{U}}_{s}-{\overset{―}{u}}_{s}}{{\overset{―}{U}}_{s}+{\overset{―}{u}}_{s}}\right],$$ (28)

$$R_{ts}^{\text{1}}={\overset{―}{T}}_{s}\left(\text{1}+e_{o}\right)exp\left[-\frac{e_{\text{1}}}{\text{2}}{\left(\text{1}+\frac{e_{\text{1}}}{\text{2}}\right)}^{-\text{1}}\right].$$ (29)

by expanding the series ${\left(\text{1}+\frac{e_{\text{1}}}{\text{2}}\right)}^{-\text{1}}$ up to one degree and ignoring the higher terms, we get


$$R_{ts}^{\text{1}}={\overset{―}{T}}_{s}\left(\text{1}+e_{o}\right)exp\left[-\frac{e_{\text{1}}}{\text{2}}\left(\text{1}-\frac{e_{\text{1}}}{\text{2}}\right)\right],$$ (30)

for bias, subtract ${\overset{―}{T}}_{s}$ and applying expectation in the equation and expanding the exponential series including the square terms, we have


$$E\left(R_{ts}^{\text{1}}-{\overset{―}{T}}_{s}\right)={\overset{―}{T}}_{s}E\left(\text{1}-\frac{e_{\text{1}}}{\text{2}}+\frac{e_{\text{1}}^{\text{2}}}{\text{4}}+e_{o}-\frac{e_{o}e_{\text{1}}}{\text{2}}\right),$$ (31)

the obtained expression for bias is


$$Bias\left(R_{ts}^{\text{1}}\right)={\overset{―}{T}}_{s}\left\{\frac{var\left(r_{\text{2}}\right)}{\text{4}}-\frac{var\left(r_{\text{3}}\right)}{\text{2}}\right\},$$ (32)

the MSE of the proposed estimator using the first order approximation may be written as


$$MSR\left(R_{ts}^{\text{1}}\right)=E{\left(R_{ts}-{\overset{―}{T}}_{s}\right)}^{\text{2}}={\overset{―}{T}}_{s}^{\text{2}}E\left(e_{o}^{\text{2}}+\frac{e_{\text{1}}^{\text{2}}}{\text{4}}-e_{o}e_{\text{1}}\right),$$ (33)

$$MSR\left(R_{ts}^{\text{1}}\right)={\overset{―}{T}}_{s}^{\text{2}}\left\{var\left(r_{\text{1}}\right)+\frac{\text{1}}{\text{1}\text{6}}var\left(r_{\text{2}}\right)-var(r_{\text{3}})\right\}.$$ (34)

### 4.2. Case-II

The exponential ratio estimator in two stage sampling for case-II scheme when $\eta_{i}=\frac{M_{i}}{\overset{―}{M}}=\text{1}$ can be defined as


$$R_{ts}^{\text{2}}={\overset{―}{t}}_{s}^{,}exp\left[\frac{{\overset{―}{U}}_{s}^{,}-{\overset{―}{u}}_{s}^{,}}{{\overset{―}{U}}_{s}^{,}+{\overset{―}{u}}_{s}^{,}}\right],$$ (35)

The proposed estimator in case-II follows exactly the same pattern as case-I. The bias and MSE for $R_{ts}^{,}$ may be presented as


$$Bias\left(R_{ts}^{\text{2}}\right)={\overset{―}{T}}_{s}^{,}\left\{\frac{var\left(r_{\text{2}}^{,}\right)}{\text{4}}-\frac{var\left(r_{\text{3}}^{,}\right)}{\text{2}}\right\},$$ (36)

$$MSR\left(R_{ts}^{\text{2}}\right)={{\overset{―}{T}}_{s}^{,}}^{\text{2}}\left\{var\left(r_{\text{1}}^{,}\right)+\frac{\text{1}}{\text{1}\text{6}}var\left(r_{\text{2}}^{,}\right)-var(r_{\text{3}}^{,})\right\}.$$ (37)

### 4.3. Case-III

The estimator for the equal units at first stage sampling units can be written as


$$R_{ts}^{\text{3}}={\overset{―}{t}}_{s}^{,,}exp\left[\frac{{\overset{―}{U}}_{s}^{,,}-{\overset{―}{u}}_{s}^{,,}}{{\overset{―}{U}}_{s}^{,,}+{\overset{―}{u}}_{s}^{,,}}\right].$$ (38)

The proposed estimator in case-III follows the similar procedure as case-I & II to obtain the bias and MSE for $R_{ts}^{\text{3}}$ as


$$Bias(R_{ts}^{\text{3}})={\overset{―}{T}}_{s}^{,,}\left\{\frac{var\left(r_{\text{2}}^{,,}\right)}{\text{4}}-\frac{var(r_{\text{3}}^{,,})}{\text{2}}\right\}$$ (39)

$$MSR\left(R_{ts}^{,,}\right)={{\overset{―}{T}}_{s}^{,,}}^{\text{2}}\left\{var\left(r_{\text{1}}^{,,}\right)+\frac{\text{1}}{\text{1}\text{6}}var\left(r_{\text{2}}^{,,}\right)-var(r_{\text{3}}^{,,})\right\}$$ (40)

## 5. Simulation study

A simulation study is conducted to evaluate the performances of proposed estimators in two stage sampling scheme using EWMA statistic followed by [23]. The comparisons of proposed estimators with existing two stage ratio and exponential estimators are done. The results of MSEs are presented in the Tables 1–4. The formula to calculate the MSEs of estimators for case-I, case-II & case-III, is

**Table 1:** MSE of two stage ratio estimators based on EWMA statistic (0.3).

**Case-I: Equal FSU and weighted mean is used**			
MSE	Proposed	Jabeen et al. (2014)	Sukhatme et al. (1984)
	27.45397	60.9272	65.02427
**Case-II: Unequal FSU and unweighted mean is used**			
MSE	Proposed	Jabeen et al. (2014)	Sukhatme et al. (1984)
	23.70502	34.18295	39.7288
**Case-III: Equal FSU**			
MSE	Proposed	Jabeen et al. (2014)	Sukhatme et al. (1984)
	17.88615	28.43498	45.91616

**Table 2:** MSE of two stage ratio estimators from normal distribution (λ = 0.5).

**Case-I: Equal FSU and weighted mean is used**			
MSE	Proposed	Jabeen et al. (2014)	Sukhatme et al. (1984)
	25.71635	64.82394	64.02527
**Case-II: Unequal FSU and unweighted mean is used**			
MSE	Proposed	Jabeen et al. (2014)	Sukhatme et al. (1984)
	19.01981	31.39109	50.09265
**Case-III: Equal FSU**			
MSE	Proposed	Jabeen et al. (2014)	Sukhatme et al. (1984)
	21.98684	27.1723	44.80206

**Table 3:** MSE of two stage ratio estimators from normal distribution (λ = 0.75).

**Case-I: Equal FSU and weighted mean is used**			
MSE	Proposed	Jabeen et al. (2014)	Sukhatme et al. (1984)
	17.49045	42.68066	42.74632
**Case-II: Unequal FSU and unweighted mean is used**			
MSE	Proposed	Jabeen et al. (2014)	Sukhatme et al. (1984)
	13.76771	32.57956	48.12345
**Case-III: Equal FSU**			
MSE	Proposed	Jabeen et al. (2014)	Sukhatme et al. (1984)
	24.07756	27.93313	67.24806

**Table 4:** MSE of two stage ratio estimators from normal distribution (λ = 0.90).

**Case-I: Equal FSU and weighted mean is used**			
MSE	Proposed	Jabeen et al. (2014)	Sukhatme et al. (1984)
	27.15109	73.35956	74.34912
**Case-II: Unequal FSU and unweighted mean is used**			
MSE	Proposed	Jabeen et al. (2014)	Sukhatme et al. (1984)
	16.79502	35.29537	36.32576
**Case-III: Equal FSU**			
MSE	Proposed	Jabeen et al. (2014)	Sukhatme et al. (1984)
	19.64737	29.10204	30.98441


$$MSE\left(y_{i}\right)=\frac{\sum_{k=\text{1}}^{\text{1}\text{0}\text{0}\text{0}}{\left(y_{ik}-\mu_{y}\right)}^{\text{2}}}{\text{1}\text{0}\text{0}\text{0}\text{0}},$$

where, $y_{i}=R_{ts},R_{ts}^{,},R_{ts}^{,,},R_{ts}^{\text{1}},R_{ts}^{\text{1}}\text{and}R_{ts}^{\text{3}}$.

The population generated from bivariate normal distribution, with $\mu_{y}=\mu_{x}=\text{2},\sigma_{y}=\sigma_{x}=\text{1}$ and *ρ *= 0.1, 0.3, 0.5, 0.7, 0.9, of size 1500 consists of 25 clusters with equal and unequal FSU’s in each cluster. Suppose that FSS, *n*, of size 4, 9 is selected then a SSS of 100 is selected from each cluster. To check the efficiency of the suggested estimator, a simulation study is designed to estimate MSE’s. We use *λ* = 0.15, 0.35, 0.55, 0.75, 0.95 for EWMA statistic.

The computed results are given in Tables 1–8, represents the MSE’s of memory type ratio estimators under two stage sampling scheme using EWMA statistic. It can be observed from the results that MSEs of the proposed memory type ratio estimators in two stage sampling are smaller than two stage ratio estimators proposed by [31] and [5]. The proposed estimators’ outperformed as compared to some present estimators.

**Table 5:** MSE of two stage ratio estimators from normal distribution (λ = 0.3).

**Case-I: Equal FSU and weighted mean is used**			
MSE	Proposed	Jabeen et al. (2014)	Sukhatme et al. (1984)
	2.37021	13.25616	4521.262
**Case-II: Unequal FSU and unweighted mean is used**			
MSE	Proposed	Jabeen et al. (2014)	Sukhatme et al. (1984)
	3.98176	20.74498	2185.346
**Case-III: Equal FSU**			
MSE	Proposed	Jabeen et al. (2014)	Sukhatme et al. (1984)

**Table 6:** MSE of two stage ratio estimators from gamma distribution (λ = 0.5).

**Case-I: Equal FSU and weighted mean is used**			
MSE	Proposed	Jabeen et al. (2014)	Sukhatme et al. (1984)
	4.74948	50.47239	8997.267
**Case-II: Unequal FSU and unweighted mean is used**			
MSE	Proposed	Jabeen et al. (2014)	Sukhatme et al. (1984)
	5.57958	22.8878	16093.37
**Case-III: Equal FSU**			
MSE	Proposed	Jabeen et al. (2014)	Sukhatme et al. (1984)
	6.46338	26.02786	3063.551

**Table 7:** MSE of two stage ratio estimators from gamma distribution (λ = 0.75).

**Case-I: Equal FSU and weighted mean is used**			
MSE	Proposed	Jabeen et al. (2014)	Sukhatme et al. (1984)
	4.08645	25.61245	6500.066
**Case-II: Unequal FSU and unweighted mean is used**			
MSE	Proposed	Jabeen et al. (2014)	Sukhatme et al. (1984)
	4.49517	17.0002	6309.153
**Case-III: Equal FSU**			
MSE	Proposed	Jabeen et al. (2014)	Sukhatme et al. (1984)
	4.80704	17.72449	18606.08

**Table 8:** MSE of two stage ratio estimators from gamma distribution (λ = 0.90).

**Case-I: Equal FSU and weighted mean is used**			
MSE	Proposed	Jabeen et al. (2014)	Sukhatme et al. (1984)
	5.97031	20.33629	4757.86
**Case-II: Unequal FSU and unweighted mean is used**			
MSE	Proposed	Jabeen et al. (2014)	Sukhatme et al. (1984)
	6.08915	20.14081	4117.297
**Case-III: Equal FSU**			
MSE	Proposed	Jabeen et al. (2014)	Sukhatme et al. (1984)
	4.46170	22.49212	9921.556

## 6. Real life application

We have used the data presented in [33] to check the efficiency of the proposed estimators. There are 7 continents considered as FSUs and 124 countries are selected according to locations from these clusters of continents as SSUs. There is only one country in the 7^th^ continent therefore; it is positioned in the 6th continent. The data of import and export in the year 1983 are considered as study and auxiliary variables respectively. Further, the data is divided into 6 clusters, say *N*, *n* = 2 and *M*_*0*_ = 124 and $\overset{―}{M}$= 20.67. The characteristics of the population data are presented in Table 9.

**Table 9:** Characteristics of the population of study and auxiliary variables.

Cluster	$M_{i}$	$m_{i}$	$\eta_{i}$	${\overset{―}{U}}_{i}$	${\overset{―}{T}}_{i}$	$S_{bt}^{2}$	$S_{bu}^{2}$	$\rho_{btu}$
1	38	15	1.8387	2254.45	1901.11	15030175.51	13586941.51	0.865
2	14	6	0.6774	25533.14	22083.21	5599280338	3256997379.00	0.989
3	11	4	0.5323	3602.82	5835.46	19221733.92	74298983.08	0.967
4	33	13	1.5968	12156.79	12438.85	526648674.70	711462797.70	0.989
5	24	10	1.1613	28493.46	30698.00	938699360.40	1599448820.00	0.506
6	4	2	0.1936	26392.50	29360.50	1814997631.0	2376032656.00	1.00

On the basis of the information mentioned in Table 9, we have calculated theMSE’s of the proposed ratio estimator for unqual FSUs and the previously develpoed estimators by [5] and [31] demonstrated in Table 10. We used different values of *λ *= 0.3, 0.5, 0.75, 0.9 to estimate the efficiency of the proposed two stage ratio estimator using EWMA statistic. In the comparison presented in Table 10, it can be observed that the proposed estimator perform efficiently than the other estimators in terms of minimum MSEs.

**Table 10:** MSE comparison of estimators.

MSE		*λ *= 0.3	*λ *= 0.5	*λ *= 0.75	*λ *= 0.9
Proposed ($R_{ts}^{,}$)	8300787	10506399	12319535	12608988
Jabeen et al. (2014)	14468635	14468635	14468635	14468635
Sukhatme et al. (1984)	74387042	74387042	74387042	74387042

## 7. Conclusions and prespectives

Two-stage cluster sampling is an essential design in large-scale surveys, and incorporating both current and past information can substantially improve estimation accuracy. In this study, memory-type two-stage ratio and exponential estimators were proposed using the EWMA statistic under three different cases of the two-stage sampling scheme. A simulation study demonstrated that the proposed estimators are efficient across different values of λ, consistently achieving minimum MSE compared to existing approaches. A real-life application further confirmed their precision and practical utility. Overall, the findings highlight that the proposed ratio estimators provide more efficient and reliable results than previously developed estimators in two-stage sampling.

Future research could extend this work by exploring memory-type estimators with alternative smoothing statistics beyond EWMA, such as hybrid or adaptive weighting schemes. Another promising direction is to investigate the performance of the proposed estimators under more complex sampling designs, including stratified two-stage cluster sampling or unequal probability selection. Applications to big data and high-dimensional survey settings may also provide valuable insights. Finally, incorporating robust estimation methods to handle outliers, measurement errors, and non-response would further enhance the practical relevance of memory-type estimators.
